# A Dictionary of Practical Medicine

**Published:** 1833-01-01

**Authors:** 


A DICTIONARY OF PRACTICAL MEDICINE, &c. &c. By *James Copland*, M.D. &c. Part I. pp. 336, and p. 16 of Appendix. Oct. 1832.

THE great Dictionary of Medicine in France, consisting of more than 60 volumes was followed successively by other works of a similar kind, though on a smaller scale, and, as was alleged, on a more careful and select plan. Something analogous appears now to be taking place in England. The Cyclopædia of Medicine, a very meritorious work, is now accompanied, rather than followed, by Dr. Copland's Dictionary of Medicine on a smaller scale, or at least in a smaller compass, but, from type and page, calculated to compress an immense quantity in a comparatively small space. We have reason to believe that Dr. Copland projected his work, and even issued a prospectus of it among his friends, before the Cyclopædia was undertaken; but the latter, from causes to us unknown, has got the start of the former. We know of no blame that can fairly be attached to either on this account. The editors of the Cyclopædia had a right to come into the field when it suited themselves, whether there was any other preparing for a similar undertaking or not—and if Dr. Copland has been anticipated in his plans, he cannot blame his more active or more fortunate cotemporaries. But, laying aside all party considerations or literary manœuvres, which are equally admissible in the cabinet as in the campaign, we believe there is ample field for both candidates in the present competition, and we are sure that the public will be gainers, whoever may be the victors.

Of Dr. Copland's talents, learning, and industry, there can be no question; and, under such circumstances, it may be a matter of doubt, or at least of discussion, which plan is preferable—that of a single compiler and compilation, or that of a cyclopædia, where each article is the work of an individual, whose name is subscribed. The former plan is the more ancient in this country—as may be seen in the Dictionaries of James and Parr; the latter is the modern mode, as exemplified in the three or four dictionaries published, or publishing, in France. Each has its advantages and disadvantages, as we said, on a former occasion, when announcing the Cyclopædia of Practical Medicine; and it would be invidious, as well as unpleasant, to dilate on the topic, now that the parties are on trial before the tribunal of the public.

From an examination of this part of Dr. Copland's work, we are led to form a most favourable estimate of its success. The labour is immense, and will stamp the author as a man of very great research, unwearied industry, and sound judgment. Like his celebrated predecessor in this walk of medical literature, Dr. Parr, the author has interwoven much of his own observation and experience, as well as his own speculative views in the various articles. It is probable that this will be considered as rather a fault; but, if we may judge by the effect on Dr. Parr's Dictionary, it will be looked on as an advantage by a much larger class of readers. The immense quantity of matter which is here compressed into a small space, must render the work a very popular one, more especially for those practitioners who reside in the country or travel abroad, on account of the facility of reference and the portability of the Dictionary, which truly deserves the name of Cyclopædia Medica. The work offers a most remarkable example of the "march of intellect"—of the facilities, as well as cheapness, of literary travelling in our days, as compared with those of our ancestors. There is more concentrated information contained in this first part of Dr. Copland's work, which costs only nine shillings, than in any five octavo volumes of the year 1800, entailing an expense of two or three guineas !!

Dr. Copland has chalked out for himself a regular systematic plan of handling his subject, which confers on the whole work a uniformity, far superior to any thing of the kind in any similar undertaking—and which is one of the advantages of being not merely editor, but author of each article.

To attempt an analysis of such an elementary work would be preposterous; and, therefore, we shall take a single article (a short one of course), and give it entire, as a sample, reserving for ourselves the opportunities that may present themselves, of remarking on particular points of doctrine or practice that may strike us, both in this work and its cotemporary, the Cyclopædia of Practical Medicine. In justice to the distinguished Editor of the Dictionary, we freely confess that the article selected is preferred on account of its conciseness, and not as affording, by any means, a favourable sample of the work. The disease which has been vaguely termed "ANGINA PECTORIS," is still unsettled in its pathology, etiology, or treatment, and, therefore, presents difficulties to a compiler of no ordinary kind; still, with all these disadvantages, we are confident that a perusal of the article will make a favourable impression on the reader, and will enable him to form an estimate of the whole. Ex pede Herculem.

ANGINA PECTORIS. SYN. *Cardiogmus Cordis Sinistri*, Sauvages. *Angina Pectoris*, Heberden. *Asthma Arthriticum*, Schmidt. *Diaphragmatic Gout*, Burton. *Asthma Dolorificum*, Darwin. *Syncope Anginosa*, Parry. *Angor Pectoris*, J. Frank. *Asthma Convulsivum*, Elsner. *Pnigophobia*. Swediaur. *Sternodynia Syncopalis*, Sluis. *Asthenia Pectoralis*, Young. *Stenocardia*, Brera. *Asthma Spastico-Arthriticum*, Stoeller. *Sternalgia*, Baumes and Good. *L'Angine de Poitrine*, Fr. *Brustbräune, Herzklemme, Bruslklemme*. Ger. *Angina di Petto*, Ital. *Suffocative Breast-pang*, Eng.

CLASSIF. 2. *Class*, Diseases of the Respiratory Function; 2, *Order*, Affecting the Lungs, their Membranes, or motive Power *(Good)*. II. CLASS, I. ORDER (*Author*, see *Preface)*.

1. Defin. *Acute constrictory pain at the lower part of the sternum, inclining to the left side, and extending to the arm, accompanied with great anxiety, difficulty of breathing, tendency to syncope, and feeling of approaching dissolution.*

2. This affection was not recognised as a distinct disease by medical authors, until Dr. Heberden described it as such in the Medical Transactions of the London College of Physicians (vols. ii. and iii.); but the works of Morgagni and Hoffmann show that they were not unacquainted with it in practice. It was also noticed by Poter (*Opera*, No. 22, p. 302.), under the head 'Respirandi difficultas, quæ per intervalla deambulantibus incidit;' and he remarks respecting it, that the attacks were sometimes so severe that persons had been suddenly carried off by them. Obscure notices of affections, which probably were of this nature in some instances, may also be detected in authors from Hippocrates downwards. From amongst these, the reader may refer to Aretæus (*Opera*, p. 7. Oxon. 1723.), Cœlius Aurelianus (lib. ii. c. i. p. 348.), Barteletti (*Methodus in Dyspnœam*, Bon. 1632.), and others, adduced by Zechinelli (*Sulla Angina di Petto*, Pad. 1813.), who supposes that the case of Seneca (*Opera*, t. ii. p. 136.), which he has himself described by the term *suspirium*, was actually this malady. Dr. Cullen has passed Angina Pectoris over in his work; but it has been well described by Drs. Fothergill, Wall, Duncan, Butter, Percival, Darwin, Macbride, Hamilton, Macqueen, Johnstone, Haygarth, Parry, Nicholl, and Good, in this country; and by Jurine, Brera, Lentin, Desportes, Kreysig, Ritter, Zechinelli, and Stoeller, on the Continent; and by Dr. Chapman, in America.

3. Pathology.—I. Symptoms. An attack of this disease is often preceded by considerable derangement of the digestive organs, especially by flatulence, acid or acrid eructations, or other symptoms of indigestion, with torpid bowels, pains in the limbs, and occasional spasms about the chest: but it frequently also attacks a patient, particularly when walking or ascending an eminence, without any, or with but slight, premonition.

4. *A.* In its *acute form*, the patient is seized with a sense of painful constriction of the chest, particularly at the cardiac region, about the lower part of the sternum, inclining to the left, and extending to the left, occasionally also to the right, arm—at first, no further than the insertion of the deltoid muscle; but the pain often successively reaches to the elbows, wrists, and sometimes even to the fingers. This is the mildest form of the disease, and soon subsides with the disappearance of its exciting cause.

5. In the more violent form of the attack, the pain and sense of constriction in the chest, and pain in the left arm, which also frequently extends to the right, amount to excruciating agony; being likened, by Laennec, to the piercing of nails or the laceration by the claws of animals. This feeling is accompanied by a sense of syncope or suffocation, sometimes with suffocative orthopnœa, convulsive dyspnœa, and palpitations; always with extreme anxiety, and a sense of approaching dissolution. The suffocative sensation is characterised by concomitant tightness and fulness of the chest, and flatulent distention of the stomach, and irritative feeling in this organ, which is relieved by eructations. During this period the pulse is variously affected, sometimes little changed, at other times extremely weak, irregular, or intermittent; and occasionally it is full, active and bounding. If the attack has been induced by walking or exercise, the patient suddenly stands still, from a feeling that perseverance in either would produce a total suspension of living power. In the slighter attacks, or early in the disease, rest merely will often immediately remove it; but this is seldom the case in the protracted and severe forms in which it frequently occurs.

6. The paroxysm continues from a few minutes to one or more hours, according to the severity and the duration of the disease. When the malady has assumed a chronic form, and its attacks occur during the night, or when the patient is at rest, the paroxysm is less violent, but generally of much longer duration; whereas, when it is induced by exertion, &c. it is of extreme violence, but of short continuance: the average duration of the fit may be about half an hour. Upon its cessation the patient merely retains a slight feeling of the various symptoms, with numbness of the arms, particularly the left. When the disease is of short standing, the paroxysms occur at long intervals, which are gradually shortened, until there is but little exemption from them, and the affection assumes a less acute character.

7. *B.* The *chronic* form of the disease is characterised by the circumstance of its being frequently a consequence of the acute; by the occurrence of the fit from the slightest causes, and after short or imperfect intervals of exemption; by its recurrence when the patient is at rest, or asleep; and by its much longer duration, but less extreme violence. Even if this form be induced by exercise, rest has little influence in shortening its duration, as in the preceding; and the paroxysm has been protracted not only for some hours, but even for several days. Palpitation of the heart, irregular and intermitting pulse, are more frequently concomitants of this state of the disease, than of the other. In the case of a very eminent and learned member of the profession, whom I long attended in this form of the disease, the attack has often continued as now described, with little remission for several weeks. Sometimes the irregularity of the pulse is observed only during the paroxysm; but in some cases it is continued, as Dr. FOTHERGILL has correctly remarked, during the intervals, particularly when they are marked by imperfect relief.

8. This form of the disease may also occur primarily. It has twice presented itself to me in this manner. During the severity of the attack, leipothymia, a feeling of dissolution from the intense agony, and these followed by palpitations, and an irregular state of the pulse, generally occur. In some cases the agonizing pain extends not only to the arm or arms, but ascends also up the throat and lower jaw, accompanied with a severe sensation of spastic constriction. In the majority of cases the above sensations are only present, when excited by motion, by assuming suddenly the erect posture, or even by attempting to read; a neuralgic kind of pain generally, however, being felt under the sternum, and extending to the arms: but in some cases, and in two which occurred to me, the exacerbations were often referrible to no very evident cause, they sometimes occurring during the night, although the above causes generally induced them.

9. Notwithstanding the remarkable distress characterising the paroxysm, this disease, particularly in its acute state, sometimes does not early affect the constitution, or entail any permanent lesion; the patient often enjoying tolerable health in the intermissions, and performing all his functions naturally, and without embarrassment, until shortly before an attack. After its protracted continuance, however, the vital energies of the frame, particularly as they are manifested in the digestive and circulating organs, give way. Marked disorder of the chylopoietic viscera, attended with various dyspeptic symptoms, occasionally with great irritability of the stomach and bowels, impeded respiration, anxious and pale countenance; flabby state of the integuments and muscles; marked derangement of the circulation, œdema, dropsy, &c.; at last supervene. But it more generally happens that the patient is carried suddenly off by a paroxysm before this state of the system is occasioned; or he sinks under the complicated derangement proceeding from an attack, and from some one of the organic changes which the continuance and repeated fits of the disease had induced.

10. II. CAUSES.—1. *Predisposing*. This disease usually attacks the middle-aged, and those beyond it; and men much more frequently than women. Of nearly one hundred cases, about seventy were upwards of fifty years of age; and seventy-nine out of the number were males; nearly one half terminated fatally, and almost the whole of them suddenly. It has been said also to occur more commonly in robust and corpulent persons with short necks. But JURINE and CHAPMAN dispute this. My own experience agrees with theirs in respect of its being equally common in persons of a spare as of a full habit. It is most prevalent in those of gouty and rheumatic diathesis, and who lead an indolent, or studious and sedentary life, or who have been subjected to much and continued anxiety and distress of mind, or indulged in much food, and spirituous or other liquors. JURINE and PARR state that they have scarcely met with it under fifty years of age. The most violent and distinctly-marked case of it which ever came before me, occurred in a gentleman at the age of thirty-four. During 1821, I attended an unmarried lady, aged twenty-six, who laboured under it in a slighter form; and recently, in 1830, another single female, at the age of twenty-five, came under my care, with the disease in its most violent grade. In both these females, it seemed perfectly unconnected with uterine disturbance, menstruation being regular, and no tendency to hysteria having at any time evinced itself, or could be detected, my attention having been directed to this point. They both ultimately recovered, after a long treatment, and the employment of very decided measures. Nearly all the cases which have come under my observation were more or less referrible to mental causes, particularly to disappointment, anxiety, and other depressing passions. Dr. HAMILTON conceives that there is an hereditary disposition to the affection. If we consider it to be of gouty origin, as contended for by Butter, Macqueen, Ritter, Stoeller, Thilenius, Elsner, and Chapman, an hereditary disposition may be also conceded. But, although very satisfactory proofs have been adduced by these authors, and particularly by Dr. Chapman, in an able paper he has recently published on this disease *(American Journ. of Med. Sciences*, No. xiii. p. 67.), yet it does not seem always to depend upon gout. Of the four cases which occurred to Dr. Black, of Newry, one only was subject to gout (*Med. Chir. Trans.* vol. vii.)

11. 2d. The *disease is usually excited* by walking, especially walking against the wind, or up hill; by ascending a flight of stairs, or any acclivity, particularly when the stomach is full or distended by flatus. It is also readily induced by either the exciting or the depressing passions, and by whatever perturbates the mind, or occasions emotion. It may also be induced by the most trifling causes, in some susceptible and irritable habits, as by gentle walking, coughing, speaking, or reading aloud; by suddenly assuming the erect posture; by straining at stool; or even by a meal, however moderate, &c. It may also occur in a state of absolute repose, particularly when the disease has become chronic; and the patient may be roused from sleep by an attack.

12. I have seen it occasioned by rapid changes of temperature, particularly by a rapid change to great cold; but different persons seem differently affected by extreme states of atmospheric temperature. In some slight cases the fit has been shortened, by the patient struggling to overcome it, by frequently attempting to make a full inspiration; but this has also failed. The patient is incapable of making this attempt in the more severe paroxysms.

13. III. Diagnosis.—Angina pectoris is more liable to be confounded with asthma, than with any other disease. But a close attention to the phenomena attending upon both affections, will readily disclose a very great difference between them. The paroxysms of asthma always come on during the night, or at the close of the day: they are characterised by a heavy dyspnœa, wheezing, and cough; are relieved by expectoration and exposure to fresh air, and subside gradually towards morning. They are not excited in the same way, nor by similar causes, nor marked by the acute and peculiar pain in the sternum and left arm, which is distinctive of angina pectoris. The stethoscope and percussion furnish us with no signs peculiar to the disease under consideration, unless it be complicated, as is sometimes the case, with organic lesion of the heart and lungs, or with effusion of fluid within the cavity of the pleura or pericardium, when they materially assist us in ascertaining the nature of the complication; and they also serve, by enabling us to ascertain other affections of the heart, to distinguish between it and them.

14. IV. Prognosis.—In recent cases, of no very violent character, recovery will frequently take place under judicious management. But when the disease has become inveterate from neglect, or from being associated with, or from having given rise to, organic lesion, and when it has appeared in a decayed constitution, or has been preceded by other diseases of the heart or lungs, an unfavourable result should be apprehended sooner or later to take place: but the period of its occurrence is uncertain; and the event is generally sudden—sometimes like an electric shock; the movements of the heart being instantly arrested. This issue is often occasioned by a full meal, or by exercise or mental emotions; but it also occurs in old or chronic cases, when the patient is at rest, and apparently uninfluenced by any circumstance or occurrence. When it is followed by symptoms of effusion of fluid within the thorax, or œdema of the extremities, a fatal termination is seldom far distant.

15. V. Proximate Cause, &c. Notwithstanding the number of examinations which have been made after death from this disease, but little light has been thrown upon it. This is not so much owing to the absence of morbid appearances, as to the extreme diversity of those which have been observed. Like epilepsy, or dyspnœa, it has presented almost every lesion, to which the organs which it affects are liable. Many of these may be viewed as accidental concomitants, or as concurrent causes; and not infrequently as results of the repeated functional disturbance occurring during repeated attacks. In several instances, not the slightest morbid appearance could be detected: but more frequently the heart and the large vessels in its vicinity have presented marks of disease, generally varied in its nature, and opposite as to its characters. The most common of these are ossification of the coronary arteries; ossification of the valves of the heart, or of the arterial trunks; enlargement of some of the cavities of the heart, either with diminished or increased thickness of their parietes; but most frequently with softening, paleness, and tenuity of the muscular structure of the organ; varicose dilatation of the coronary veins (BRERA); depositions of adipose matter, to the extent of impeding its functions; effusions of serum, blood, &c. into the pericardium or cavity of the pleura, &c. (FOTHERGILL, BLACK, &c.) It has justly been remarked, by my friend Dr. UWINS, 'that there is scarcely any malformation of the heart, or its blood-vessels, that has not been occasionally found after death, from what would be considered angina pectoris: while, on the other hand, individuals have fallen victims to the affection, fully marked, and the most accurate post-mortem examination has not been able to detect the slightest indications of structural derangement.' *(Compend. of Theoret. and Pract. Med.)* In some cases, the only morbid appearances observed have been in other, and distant organs, from that which seems to be, if not the chief seat of the disease, at least the organ chiefly affected in its functions by it—the heart and large vessels having been altogether exempt from lesion. These appearances were adhesions of the serous surface of the lungs to adjoining parts; serous effusions into the pleura; thickening of the respiratory mucous surface; dilatation of the bronchi; œdema of the intervesicular cellular tissue of the lungs; abscess and tumours in the mediastinum; ossification of the cartilages of the ribs (WICHMANN, JAHN); tubercles, enlargement, scirrhosity, &c. of the liver (PERCIVAL, LATHAM, BRERA, and WALKER); scirrhus of the pylorus, &c.

16. These lesions serve less to throw light on the precise nature of the disease than an attentive examination of the morbid phenomena during the life of the patient, and a calm appreciation of their relations, particularly with respect to the agents tending to diminish, remove, or to exasperate them. This affection has been considered by many authors as spasmodic, 'although the part immediately concerned seems not to have been designated or understood.' Dr. CHAPMAN remarks, that this hypothesis is rendered probable, by the general complexion of the disease—its causes, symptoms, and cure—and by its analogy to other disorders confessedly of this character.

17. Dr. FOTHERGILL supposed it to be occasioned by obesity, and particularly by a collection of fat about the heart; he also considered that it was sometimes symptomatic of water in the pericardium or cavity of the thorax. PARRY, JENNER, BURNS, KREYSIG, BOSTOCK, and some others, have viewed this affection as a species of syncope occasioned by the accumulation of blood in the heart, from an ossification of the coronary arteries. Drs. HOSACK and FORBES conceive that it most frequently arises from a plethoric state of the blood-vessels, more especially from a disproportionate accumulation of blood in the heart and large vessels. To the first and second of these opinions it may be objected, that there is no obvious connexion between the effect and the cause; for, as the cause is permanent, the effect should be continued, or at least present but little abatement, whereas the intermissions between the paroxysms are often characterized by a return of the healthy functions. It may be further stated, in opposition to this hypothesis, that many fatal cases have occurred in which this particular lesion was not found on dissection. LAENNEC states that he has examined several subjects who had laboured under this disease, and in none of them did he find the coronary arteries ossified. Besides, cases are recorded by MORGAGNI, SENAC, WATSON, CORVISART, ANDRAL, and others, in which ossification of these vessels was not productive, during life, of the sufferings characterising this disease. Indeed the coronary arteries are often found ossified in old persons, who had not complained during life of any affection of the heart, and who certainly never were attacked by this malady. As to the last of the above opinions, viz. that adopted by Dr. HOSACK, Dr. CHAPMAN has very justly observed, 'that even allowing the fulness and irregularity of the circulation contended for, which I am by no means disposed to do, as uniform concomitants, these I should take to be rather the effects of previous irritation or excitement, than the cause of the disease. Do we not also know, that such a condition of the vessels can exist without inducing angina pectoris? Were fulness and irregularity in the circulation only required for the production of the disease, instead of a rare, would we not have it as a daily occurrence? The fact, moreover, is, that angina pectoris, though oftener, perhaps, attacking the plethoric, is to be met with, as I have before said, in the feeble and attenuated.' I may add to this, that the severest case of the disease which has ever occurred to me was that of a gentleman who had suffered severely from repeated and profuse hæmoptysis, and other symptoms of disease of the lungs. All these disappeared, but were followed, after some time, by angina pectoris. He was feeble and attenuated; but it was considered advisable to try the effect of blood-letting to a moderate extent: this gave no relief; it was repeated, but the symptoms were evidently aggravated by the measure.

18. Dr. JURINE considers the disease as a nervous affection; and he supports this opinion by referring to the sudden and unexpected manner of its attack—to its sudden termination in death, or restoration to health—the nature of the exciting causes of the paroxysm—the equality and regularity of the pulse, in the majority of cases, during the paroxysm—to the state of the respiration—to the painful sensation extending to the upper extremities—and lastly, to the circumstance of antispasmodics being beneficial in its treatment. The proximate cause, he adds, consists of an affection of the pulmonary nerves, disturbing the functions of the lungs, impairing the decarbonisation of the blood, and producing the pain in the sternum. This affection of the pulmonary nerves is communicated to the cardiac plexus, and deranges, secondarily, the heart and large vessels. The imperfect decarbonisation of the blood diminishes its stimulating influence on the heart and lungs, giving rise to repeated attacks, until it occasions the death of those organs, and then of the brain.

19. MM. DESPORTES and LAENNEC have adoped a nearly similar view of the disease, with this difference, that they consider its particular seat may vary according to circumstances. Thus, M. LAENNEC states, that when there exists, simultaneously, pain in the heart and lungs, we may presume that the affection is seated chiefly in the pneumogastric nerves; but where there is simply stricture of the heart, without pulmonary pain or difficulty of breathing, its site is in the nerves which the heart receives from the great sympathetic. But he supposes that other nerves may also be implicated at the same time, either by direct anastomosis or by sympathy; and that the branches of the bronchial plexus, particularly the cubital, are nearly always so affected. 'The anterior thoracic originating in the superficial cervical plexus are, moreover, frequently implicated; and this is sometimes further the case with the branches derived from the lumbar and sacral plexuses, when the thigh and leg participate in the attack, which occasionally happens.'

20. BRERA, ZECHINELLI, AVERARDI, and some others, consider the disease to be occasioned by pressure of enlarged abdominal viscera on the heart, particularly of enlarged liver. JOSEPH FRANK conceives it to proceed from congestion of the cavities of the heart, occasioned by defective nourishment of its muscular structure; this defective nutrition itself resulting from previous inflammation, or from metastasis of gout or rheumatism, or from disease of the coronary arteries. (*Prax. Med. Univ. Precep.* t. ii. p. 260.) Respecting these, it may only be added, that the symptoms of angina pectoris are very seldom associated with enlargement of the abdominal viscera; and that, although they are much more frequently connected with the lesions alluded to by FRANK, this connexion is by no means uniform, and is obviously not one of cause and effect; these lesions being rather coincident and partial results of the morbid state of the nerves, the altered sensibility of which constitutes one of the chief characteristics of the disease. It may he farther stated, that Dr. DARWIN views it as a particular species of asthma, producing cramp of a peculiar kind in the diaphragm, or the other muscles of respiration; and Dr. BUTTER, while he conceives it to be of gouty origin, also refers it to the respiratory organs, particularly to the diaphragm. On these opinions it is unnecessary to comment.

21. Dr. CHAPMAN, to whose valuable paper I have already referred, states, 'That the disease is a species of neuralgia, I am entirely persuaded, commencing for the most part in the pneumo-gastric nerve, and spreading in different directions, as other nerves may become involved. The derangement of the heart and other structures, with which it is sometimes associated, I hold to be coincidences or effects, and not the cause; since, among many reasons which might be adduced in corroboration of it, the disease has undoubtedly prevailed independently of such organic lesions, and, conversely, these have existed without occasioning it. But what is the immediate cause of the irritation of the nerves, inducing this neuralgic condition, giving rise to the subsequent phenomena of the disease? This is a question, which hitherto has not been clearly answered. My conviction is, that it is derived from irregular gout, which misplaced, thus operates as an irritant of the nerves, and probably first of those of the stomach.'

22. It will be remarked from the foregoing, that JURINE, DESPORTES, LAENNEC, and CHAPMAN agree so far as to impute the disease to a species of neuralgia of the pulmonary and cardiac nerves, affecting the functions of the heart and respiratory organs, and extending by nervous connexion to other parts; the organic lesions found in fatal cases being either coincidences, or effects of the disease; and after an attentive examination of the phenomena attendant on several cases of the affection which have come before me, I see no reason for differing materially from this opinion. With regard to the origin of this affection of the nerves in misplaced gout, I cannot so implicitly agree with Dr. CHAPMAN. The connexion had been previously remarked by several physicians, as I have already stated, particularly by those whose names have been adduced, as well as by SCHMIDT and BURTON,—a circumstance favourable to the idea that it is founded in truth; and evidence of it may even be found in Dr. MUSGRAVE's very excellent, but now scarcely ever noticed work, on Anomalous Gout. WICHMANN, however, has disputed this connexion, and apparently with much reason. The notice which had been taken of this morbid relation is very candidly referred to by Dr. CHAPMAN, who has adduced the particulars of six cases in which this affection was evidently connected with gout, and in which recovery took place, after means had been successfully employed to invite this disease to the extremities. In the majority of those cases the patients had never previously suffered a gouty attack, and yet the means employed were successful in causing it to appear in the lower extremities.

23. But whether this disease is merely a form of misplaced gout, or an affection *sui generis*, which, when occurring in persons of a gouty diathesis, the induction of the regular gouty paroxysm in the extremities generally removes, my experience does not enable me to decide. In two persons whom I was lately called to treat, and with whom I have been long acquainted, I have no reason to suspect a gouty tendency; but the connexion so satisfactorily established by Dr. CHAPMAN is evidently by no means infrequent, and is one which ought never to be overlooked during the treatment of this most distressing and dangerous disease. I believe that, in addition to the nervous character of the malady, the substance of the heart is often weak, thin, pale, and attenuated, or even softened, as if its substance were imperfectly and unhealthily nourished; and that its cavities, consequently, become occasionally dilated and congested. This view is accordant with the treatment generally found most successful in removing it. In a great proportion of the cases before referred to (§ 10.), of which I had made notes, chiefly collected from authors, dissection had been made in about fifty of those which were fatal; and out of this number nearly forty presented some degree of disease of the heart or large vessels;—most frequently ossification of the valves, coronary arteries, and aorta; and softening and emaciation of the heart. But whether these lesions were rather the consequence than the cause of the disease may be disputed.

24. VI. The TREATMENT of this disease necessarily respects, 1st, the measures which may be adopted during the paroxysm; and, 2d, those which should be resorted to in the intervals, with the view of effecting a perfect cure.

25. 1st, *In respect of the means which may be employed during the fit, with the view of diminishing its duration and violence,* no very precise or dogmatic direction ought to be given. Much will depend upon the peculiar characters of the case. The patient should always be placed in a state of tranquillity; and, particularly, if the countenance be pale, and the carotids pulsating feebly, in the supine or reclining position. The propriety of *bleeding* in the fit has been discussed by several physicians, and depends entirely upon the particular features of the attack. Where the symptoms are urgent, the patient plethoric or vigorous, or the pulse full and possessed of tone, there can be no doubt as to the propriety of the measure. Dr. READ (*Dub. Med. Trans.*, vol. i. p. 105.) has recorded a case which well illustrates the good effects of this treatment during the paroxysm. In more questionable cases, where the pulse is weak, and the countenance is collapsed, bleeding from the arm ought not to be had recourse to. It is doubtful whether or not cupping even should be employed; but where this latter state is not extreme, and especially in cases of intermediate grades of severity, cupping between the shoulders, to a small or moderate extent, as the case may seem to require, will generally afford relief, particularly if used simultaneously with derivatives to the extremities.

26. But in nearly all cases, and still more particularly in those characterised by syncope, and an imperfect action of the heart, *frictions* with stimulating and irritating substances ought to be previously employed over the anterior parts of the thorax, and *stimulants* and *antispasmodics* exhibited internally. As to the extent and repetition of the blood-letting, whether general or local, the practitioner ought to be able to decide, being guided in this, as in other remedial means, by the apparent energies of the constitution, and the state of the vascular system; if these admit, and especially if signs of plethora, or of congestion of the cavities of the heart and large vessels of the chest exist, the depletion may be carried to a considerable extent, or repeated, according to the relief obtained. The object here is to reduce the body to be moved to a nearer relation to the state of the moving power, at the same time that we endeavour to increase the energy of the latter.

27. I should add, that the propriety of bleeding, in the paroxysm particularly, has been much disputed; and especially by Continental authors. Where the pulse is feeble and soft, and the action of the heart weak, it is generally inadmissible; but, wherever we entertain doubts respecting it, the external and internal use of stimulants and antispasmodics, with frictions, should be cautiously premised, and only local depletions adopted; or depletion of every kind should be entirely omitted until after the paroxysm, when either general or local blood-letting, according to the particular circumstances of the case, may be practised with necessary precautions. I have employed moderate blood-letting in three cases, in which the propriety of the measure seemed questionable, the patients being of spare habits of body, and weakened states of system; but every precaution was taken to prevent immediate ill effects from the operation. In one of the three relief was afforded; in another, the advantage was very doubtful; and, in the third, the disease was evidently exasperated by it, although slight benefit seemed to result from it at the time. In one of those cases the serum of the blood had a milky appearance, from the presence of an oily matter, resulting from imperfect assimilation. From this evidence, therefore, I infer, that, where there are no signs of vascular plethora or cardiac congestion, or where the vital energies of the patient are depressed, and we presume the substance of the heart is attenuated and imperfectly nourished, we should be extremely circumspect in having recourse to vascular depletions of any description, and should particularly avoid bleeding from a vein; but, at the same time, we should be equally careful not to administer too active stimulants.

28. Next to the employment of depletion, under the above restrictions, in suitable cases, and with the concomitant means recommended, the bowels may be opened by a *purgative medicine*, combined with some warm *antispasmodic* and *carminative*, as ether, spiritus ammoniæ aromaticus, camphor, musk, castor, spiritus anisi, &c.; and these may be given, at intervals, subsequently. In the slighter attacks, and where the state of the vascular system and constitutional energies render it prudent to withhold depletion, friction, with stimulating liniments over the thorax and epigastrium, (as the following:—

No. 14. ℞. Linimenti Camphoræ Comp., Linim. Ammoniæ fort., aa ℥j.; Tinct. Capsici, ʒiij. M.)

the internal administration of antispasmodics, and the exhibition of a purgative medicine, will be sufficient to give some immediate relief. The following will generally fulfil the intention:—

No. 15. ℞. Infus. Valerianæ, ʒxj.; Spirit. Ammoniæ Fœtid. ʒss.; Tinct. Castorei, ʒss. M. Fiat Haustus bis terve in die capiendus.

No. 16. ℞. Infus. Sennæ Comp. ℥jss.; Tinct. Sennæ, ʒij.; Spirit. Ammon. Arom. ʒss.; Tinct. Cardamom Comp. ʒj. M. Fiat Haustus statim sumendus, et repet. si sit occasio.

Or the following:—

No. 17. ℞. Mist. Camphoræ, ℥j.; Liq. Ammon. Acet. ʒij.; Spirit. Æther. Sulph. Comp. ʒj.; Tinct. Camphoræ Comp. ʒj.; Syrup. Papaveris, ʒj. M.

29. *Emetics* have been spoken favourably of by Dr. Good (*Study of Med.*, t. i. p. 667.) In a case of great severity, in which vomiting occasionally occurred when the paroxysm was excited by taking food into the stomach, I was induced by this symptom to try the effect of an emetic during an attack, but no benefit was derived from it.

30. The employment of *derivatives* to the extremities, particularly the lower, is generally beneficial; and ought not to be omitted in the paroxysm, whether we adopt the opinion as to the gouty origin of the disease or not. *Stimulating pediluvia*, and *sinapisms* or *blisters*, with all the other measures employed under similar circumstances in irregular or misplaced gout, had the effect, in the six cases of the disease published by Dr. Chapman, of inducing the regular gouty paroxysm, and of affording speedy relief. The *affusion of cold water* has been recommended by some authors, but it is a dangerous remedy in this disease. *Cold epithems* to the head have been mentioned by J. Frank (*Prax. Med. Univers.*, part ii. p. 273.), as having been used with advantage; they seem less objectionable. A similar remark may be applied to the tepid affusion on the head.

31. 2d, *The means which may be employed during the intervals or remissions between*

*the paroxysms* are either *general* or *topical*. With respect to the *first* of these, a most studious attention to avoid the exciting causes of the disease must be inculcated. Next to this, all existing disorder of the digestive organs should be attended to and removed; and the diet and regimen of the patient strictly laid down and enforced. As the powers of the digestive organs are generally diminished, and the bowels either costive or irregular, *vegetable bitters*, with an occasional alterative aperient, either given alone, or in combination with an antispasmodic or anodyne, will often prove beneficial. With the view of thus strengthening the digestive organs and removing spasm, SCHÆFFER (*Volkskrankheiten*, Jun. 1807.) recommended vegetable bitters with opium, musk, camphor, or assafœtida, and ELSNER prescribed the *muriate of ammonia* with Hoffman's anodyne. *Sulphate of zinc*, recommended by PERKINS (*Mem. of Med. Soc. of Lond.* v. iii.) in doses of a grain, with a quarter of a grain of opium, given twice a day, has a similar action; but it generally is necessary to give it more frequently, and to increase the doses. With the same view I have given the *prussic acid*, either simply, or combined with the oxide of zinc, forming a *prussiate of zinc*, and in one case particularly, with greater advantage than from any other means. I have reason to believe that the *prussiate of iron* will prove equally beneficial; but my experience of its effects is too imperfect as yet to allow me to speak decidedly as to its merits in this disease.

32. In a case which occurred to me a year since, I employed the *preparations of iron*, particularly the carbonate, being led to adopt them by the neuralgic characters of the case, and certainly with apparent advantage; but I should add, that local means were also in operation at the same time. Wherever we have reason to suppose that the heart is debilitated, imperfectly nourished, or attenuated, the employment of tonics, particularly bark, and the preparations of iron, either alone or with antispasmodics, is particularly indicated, with strict attention to diet and regimen. *Auscultation* will be found of service, by intimating to us the particular state of the heart, which must in a great measure regulate our practice.

33. In a case of the disease which came under my care in 1824, I prescribed the *nitrate of silver* triturated with a vegetable extract, as recommended by SEMENTINI. This substance was continued in increased doses, until it occasioned an eruption, resembling nettle-rash, on the skin,—an effect noticed by this physician. The relief afforded by it, after this eruption began to appear, was decided. The patient is, at the present time, in the enjoyment of tolerable health. At the period of my prescribing this substance, I conceived that its exhibition in this disease had originated with myself; but I subsequently found that it had been given in two cases of angina pectoris, with advantage, so long ago as thirty years, by Dr. CAPPE (*Duncan's Annals of Med.*, vol. iii.).

34. *Arsenic*, in the form of Fowler's solution, had been recommended in this disease by Dr. ALEXANDER (*Med. Comment.*, vol. xv. p. 373.), at a period antecedent to the introduction of the nitrate of silver into practice, as an internal medicine; and subsequently by Sir G. BLANE, who gave it with advantage, combined with digitalis and mercury (*Med. Chir. Trans.*, vol. iv. p. 136.).

35. Besides these, preparations of *bark*, and other vegetable tonics, have been recommended, either alone, or in combination with antispasmodics and anodynes. The *hydrosulphuret of ammonia*, in gradually increased doses (from eight drops to thirty) twice or thrice daily. The different preparations of *valerian*, the *cuprum ammoniatum*, and *sulphate of quinine*, have likewise been employed, and occasionally with decided advantage: from the last of these, combined with an anodyne, particularly with opium and camphor, I have observed much benefit to be derived. The following formulæ may be employed.

No. 18. ℞. Infus. Rosar. Co. ℥xj.; Quininæ Sulph. gr. j.—ij.; Acidi Sulph. Arom. ♏ x.; Spirit. Æther. Sulph. Comp. ʒj.; Tinct. Opii, ♏xij. M. Fiat Haustus bis in die capiendus. Or,

No. 19. ℞. Extract. Anthemid. ℈ij.; Quininæ Sulph. gr. xij.; Massæ Pilul. Galban. Comp. ℈j.; Camphoræ Subactæ, gr. xv.; Syrup. Papaveris, q. s. Misce benè et divide in Pilulas, xxiv., quarum capiat unam ad binas vel tres bis terve quotidiè.

Having derived much advantage from the internal use of the *sub-borate of soda* in dyspeptic irritability of the alimentary canal, I was induced to employ it in a case of this disease which occurred to me a few years since, in doses of from twenty to thirty grains, given in the decoctum althææ. It produced some relief; but the case was of the greatest severity, and little benefit, at least of a permanent description, was derived from any means which were adopted, excepting from the prussic acid.

36. *Mercurials* have received the sanction of Brera. I have employed them in two cases: at first as an alterative; five grains of blue pill having been directed occasionally at bed-time, and subsequently so as to affect the mouth. In one of these the alterative dose had a beneficial effect upon the state of the stomach and bowels; but this was of short duration. When, however, pushed further, so as to affect the gums, great irritability of the system, fever, restlessness, and increased pain, anxiety, and sinking, were occasioned by it. In the other case, evidently connected with hepatic disorder, the blue-pill was also at first given as an alterative, on alternate nights. It affected the gums after a few doses, and afforded relief. It was now pushed with the intention of inducing salivation; and a somewhat violent effect was produced on the mouth, which was relieved upon exciting the salivary glands. Decided advantage was now procured; the bowels were kept open by means of a stomachic aperient, an issue inserted in one of the thighs, and change of air recommended. This patient perfectly recovered.

37. Where plethora exists, *blood-letting* in the intervals will be serviceable, with a light abstemious diet. When the paroxysms are apt to occur during the night, I have found an opiate given at bed-time, as recommended by Dr. Heberden, of great service. In one case of this description I gave the *acetate of morphine*, in the dose of an eighth of a grain, but it occasioned such distressing feelings of sinking, and general depression of the powers of life, that stimulants were required; yet the same patient had experienced relief from opium combined with camphor. On one occasion I tried the effects of *iodine* in the form of the tincture; but, although its use was adopted with great caution, seven drops only having been given three times a day, it occasioned an increase of all the symptoms, apparently owing to its irritating effects on the digestive mucous surface, and the idiosyncrasy of the patient. I may here notice the practice recommended by Schlesinger (*Hufeland's Journ.*, vol. i. p. 57.), consisting in the exhibition, every two hours, of the extract of the *lactuca virosa*, in doses of two grains, with half a grain of *digitalis*. What effect may we expect from the use of *colchicum?* Where the disease seems to originate in gout, the colchicum might be tried; but its use would require great circumspection. In my opinion it should only be given in combination with stimulants, or antispasmodics and tonics, the spiritus colchici ammoniati being the most promising preparation of it in such a case.

38. Although the patient labouring under this disease is generally incapable of any, excepting the most gentle, exercise; yet this should be taken under favourable circumstances; and change of air, particularly to healthy, dry, and elevated situations, should not be overlooked. It will generally be observed, that persons labouring under the worst form of the disease, incapable even of walking or sitting upright for any time, will bear well, and even be benefited by, rapid travelling in a carriage. This was first evinced to me by the case of a gentleman of great scientific and literary attainments, residing for a time at Paris, where I was called to him in the summer of 1829. He was anxious to return to England, from a dread of dying abroad. He undertook the journey with me, and was better during it than either previously or subsequently. He has since taken long journeys, with similar advantage, but no means which have hitherto been employed have afforded him more than temporary relief.

39. *Secondly*, Much benefit will be often received from *topical* means. Under this head *issues* and *setons* deserve particular notice. They have been employed on the insides of the thighs by Macbride and Darwin. Kreigelstein and Wolff also have observed advantage to be derived from them, when inserted either in this or in other situations. I have resorted to a peculiar form of issue in several cases of this disease, and, upon the whole, with much benefit. In one case, however, it failed of having the least good effect.

40. The *form of issue* to which I allude, and for the knowledge of which I am indebted to my learned friend Dr. Hutchinson, is the bark of mezereon root, deprived of its external cuticle, and, after having been soaked for some time in a little water, placed upon the surface of the part from which we wish to procure a discharge. This bark should be confined to its place by means of adhesive plaster, spread on paper of larger dimensions than the part covered by the mezereon bark. The bark may be renewed every night, until it procures a copious discharge. In some cases the effect is produced in a single night, or in twenty-four hours. When the discharge becomes copious the bark may be renewed less frequently. The adhesive plaster serves both to keep the mezereon in its situation, and to retain the discharge, so as to preserve it from soiling the clothes. When it is abundant the plaster may be renewed, and the secretion removed, as its occasional acrimony often tends to heighten and to extend the irritation. In a severe and chronic case of this disease, which occurred to me lately (in 1830), I employed this form of issue, and kept a surface of about four inches square over the left small ribs discharging as long as the patient would endure this treatment. The disease disappeared, and up to this time it has not returned. The advantages of this issue are, that the patient can manage it from the beginning with great ease; and it may be readily increased to any extent, and the discharge augmented, according to the exigencies of the case.

41. *Artificial eruptions*, from the *tartar emetic* ointment or plaster, have now usurped the place of setons and issues; but, from a very extensive experience of the former, both previous and subsequent to the publication of an article on them in the London Medical Repository for April 1822, I consider them of inferior efficacy in some diseases, and particularly in this, to the pea-issue, or the issue now described. It is singular that the advantages to be derived from the production of artificial pustulation, in the treatment of various disorders, were so little known or appreciated until the appearance of Dr. JENNER's pamphlet on the subject, since the practice had been recommended long previously in the Lectures of the second and third MONROS on Morbid Anatomy, as being frequently preferable to the use of blisters; and had been found serviceable by GOODWIN, AUTENRIETH, and KREIGELSTEIN, in this affection, in which it had been employed by them at the end of the last century.

42. *Blisters*, either frequently repeated, or kept discharging for a longer or shorter period, have received the sanction of PERCIVAL and many others. But little benefit will be derived from them, unless they be used in the way now named. THILENIUS recommends (*Med. und Chir. Bemerkungen*, i. p. 183.) repeated blisters applied between the shoulders. I agree with him in the selection of this place in preference to others for their application, as well as in the propriety of repeating them frequently. M. LAENNEC states that he has derived great advantage from *magnetism*, used in the following manner, both in alleviating the paroxysm and in preventing its accession:—He applies ' two strongly magnetised steel plates, of a line in thickness, of an oval shape, and bent so as to fit the part,—one to the left præcordial region, and the other exactly opposite, on the back, in such a manner that the magnetic current shall traverse the affected part.'—(*Diseases of the Chest*, p. 705.)

43. When the affection is *complicated* with other diseases, particularly with organic lesions of the heart, or enlargement of the liver, the treatment should be modified accordingly. In order to ascertain the nature of such complications, auscultation may be resorted to; for, although it gives us no information respecting the simple disease, it often enables us to detect the lesions with which it is sometimes associated, and to direct our means of cure more appropriately, and with happier results, than we could otherwise do. When the substance of the heart is weakened or attenuated (§ 23.) tonics, particularly sulphate of quinine, sulphate of zinc, and the various preparations of iron, given in decided doses, are particularly indicated. In other cases, as well as when the liver is affected, issues are generally serviceable. When the disease is connected with enlargement, &c. of the liver, mercury is almost indispensable. In all cases, whether simple or complicated, attention to diet and regimen, a pure air, amusement without excitement, and an equable and contented state of mind, are not only requisite to recovery, but are also necessary to render it permanent.

BIBLIOGRAPHY.—*Sauvages*, Nosologia Methodica, tom. iv. p. 120. edit. 8vo.—*Heberden*, Medical Transactions of the College of Physicians, vol. ii. p. 59, 1768.—*Elsner*, Abhanlung über die Braustbraune. Kœnisb. 1778.—*Schæffer*, Dissertat. de Angina Pectoris. Gotting. 1787.—*Butter*, Treatise on the Disease commonly called Angina Pectoris. Lond. 1791.—*Schmidt*, Dissert. de Angina Pectoris. Gott. 1793.—*Parry*, An Inquiry into the Symptoms and Causes of the Syncope Anginosa. Lond. 1799.—*Hesse*, De Angina Pectoris. Halle, 1800.—*Darwin*, Zoonomia, vol. iv. p. 42. 1800.—*Stoeller*, Journ. der Pract. Heilkunde von *Huffeland*, 17 b. 1803.—*Jahn*, Ueber die Syncope Anginosa, (Huffeland's N. Journ.) 1806.—*Beaumes*, Traité Elementaire de Nosologie. 1806.—*Desportes*, Traité de l'Angine de Poitrine. 1811.—*Blackal*, Observations on the Nature and Cure of Dropsies, &c. Lond. 1813.—*Kreysig*, Die Krankheiten des Herzens. 8vo. Berl.—*Zechinelli*, Sulla Angina di Petto, Pad. 1813.—*Jurine*, Mémoire sur l'Angine de Poitrine, couronné par la Societé de Medicine de Paris. 1815.—*Laennec*, Traité de l'Auscultation Mediate. Paris, 1826.—*Chapman*, American Journal of Medical Sciences, vol. vii. Phil. 1831.—*Jolly*, in Dictionnaire de Medicine et Chirurg. Pratiques, &c. tom. ii. Paris, 1829.

When we inform our readers that the above article occupies only *seven pages and a half* of the Dictionary, he will be able to form some estimate of the value and the extent of the matter contained in this first part, as well as of the plan or arrangement on which Dr. Copland proceeds. It is now universally allowed that competition is the soul of enterprize and improvement, in literature and science, as well as in the mechanical arts. We are not sorry, therefore, to see two competitors (we will not designate them rivals) in the field of medical lexicography. They will benefit each other, and the public generally will be benefited. There is ample market for both publications. Many will possess themselves of both—and there are two large classes, each of which make a selection, some taking in the one, and others the other. We heartily wish the competitors success in proportion to their merits, and if they obtain that, they will have good cause to be satisfied.

In this first part, there are several very important articles, especially those on apoplexy, asthma, blood, and affections of the brain. The latter is a monograph of great merit, and immense concentration of scattered information. The diseases of the bronchi and air-passages are developed in a very masterly manner, and do great credit to the erudition and discernment of the editor. In fine, this first sample of the work will insure it success—and prove an earnest of Dr. Copland's literary reputation.

